# Porpoise Flesh as Food

**Published:** 1885-05

**Authors:** 


### Porpoise Flesh as Food.

Porpoise fishing promises to become one of the principal industries on the New Jersey coast. At first porpoises were caught for the skin and blubber alone, the carcasses being thrown away or, in a few instances used as fertilizers. From the blubber is extracted a very fine oil, while the skin makes a superior quality of leather. Some time last fall it was discovered that porpoise flesh was savory to the taste, and it soon became popular as an article of food. The juicy red meat, which is taken from beneath a layer of fat next to the skin, is pronounced by epicures to be more palatable than any porter-house steak ever cooked. In appearance it resembles beef, but is more solid and of finer grain and very tender. Some persons say that it tastes like venison, and that there is nothing of the fishy taste about it. Last fall New York fish dealers offered two cents per pound for the carcasses, but they will command a much higher figure next summer. The fishermen have received orders from prominent Philadelphia and New York hotels and restaurants, and it is believed that there will be a demand for the meat which cannot be met. In addition to this, much of it will be dried and placed upon the market. It is superior to dried beef, as in this state it retains all of its sweetness, and is still as tender as when fresh. The porpoises weigh from three hundred to four hundred pounds, and are caught in a seine, which is necessarily large and cumbersome, as the fish are very powerful when they are in the water. Last year a seine about 1,000 yards long, with a net reaching almost to the bottom of the sea, was used, but it was seldom that more than half a dozen fish were taken in a haul. Improved seines will be used this season. Each porpoise was estimated to be worth $20 when nothing but the blubber and skin were used, but now that the flesh is in demand and extra fine oil is extracted from the heart, it is said that they will be worth more than double that amount.—*Boston Transcript.*

[To the above uses to which the porpoise is put might be added, the manufacture of watch-makers oil. This oil is made from the skull of the porpoise. It is extremely valuable, being the only oil which will not harden and clog up the delicate mechanism of a watch.

The manufacture is confined to one firm, if we remember rightly, and the price is enormous. Still, an ounce of the oil will last a watchmaker many years.

The leather made from porpoise skin is extremely soft and pliable, and is very strong. It is, however, too elastic for shoe leather, soon losing its shape. Owing to its durability and strength, it is used for shoe laces, for which purpose it has no equal.—Ed.]

